# Group Selection as Behavioral Adaptation to Systematic Risk

**DOI:** 10.1371/journal.pone.0110848

**Published:** 2014-10-29

**Authors:** Ruixun Zhang, Thomas J. Brennan, Andrew W. Lo

**Affiliations:** 1 MIT Department of Mathematics, Cambridge, Massachusetts, United States of America; 2 Northwestern University School of Law, Chicago, Illinois, United States of America; 3 MIT Sloan School of Management, CSAIL, and EECS, Cambridge, Massachusetts, United States of America; 4 AlphaSimplex Group, LLC, Cambridge, Massachusetts, United States of America; CNRS, France

## Abstract

Despite many compelling applications in economics, sociobiology, and evolutionary psychology, group selection is still one of the most hotly contested ideas in evolutionary biology. Here we propose a simple evolutionary model of behavior and show that what appears to be group selection may, in fact, simply be the consequence of natural selection occurring in stochastic environments with reproductive risks that are correlated across individuals. Those individuals with highly correlated risks will appear to form “groups”, even if their actions are, in fact, totally autonomous, mindless, and, prior to selection, uniformly randomly distributed in the population. This framework implies that a separate theory of group selection is not strictly necessary to explain observed phenomena such as altruism and cooperation. At the same time, it shows that the notion of group selection does captures a unique aspect of evolution—selection with correlated reproductive risk–that may be sufficiently widespread to warrant a separate term for the phenomenon.

## Introduction

Since the publication of the path-breaking work of Wynne-Edwards [1] and Hamilton [2], [3] in the 1960s, the theory of evolution has been applied to much broader contexts than self-replicating genes. By appealing to notions of inclusive fitness and kin and group selection, compelling explanations for previously inexplicable behaviors such as altruism and cooperation have been developed. This approach has generated a number of additional insights such as reciprocity [4], the Price equation [5], sociobiology [6], [7], and the theory of multi-level selection [8]. Moreover, empirical studies have lent further support to the theory of group and kin selection, including social behavior in bacteria [9]–[11], sterility in social insects [3], [12], and the avoidance of cannibalism in salamanders [13].

The behavioral implications of group selection have also received considerable attention from economists, who have used evolutionary models to explain apparent conflicts between individual rationality and human behavior [14], [15], including attitudes toward risk and utility functions [16], [17], time preference [18], and financial markets [19], [20]. As an alternative to the traditional view that “market prices fully reflect all available information” [21], [22], the Adaptive Markets Hypothesis [23] provides an evolutionary interpretation of financial market dynamics.

Despite these applications, group selection is still one of the most hotly contested issues in evolutionary biology. An enormous body of research has been dedicated to understanding the relationship between genetic, organismic, and group selection [24]–[26], and the relationship between group and kin selection [27]–[32]. Critics over the last few decades have argued forcefully against group selection as a major mechanism of evolution, and recent attempts to revive it [30], [33], [34] have been met with swift and broad rebuke [35], [36].

Here we propose a reconciliation of the two opposing perspectives by arguing that what appears to be group selection may, in fact, simply be the consequence of natural selection occurring in stochastic environments with reproductive risks that are correlated across individuals. Those individuals with highly correlated risks will appear to form “groups”, even if their actions are, in fact, totally autonomous, mindless, and, prior to natural selection, uniformly randomly distributed in the population.

We illustrate our approach with the following simple example. Consider a population of individuals, each facing a binary choice between one of two possible actions, 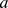 and 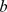, and suppose the environment consists of two possible states of nature, rain or sunshine, with probability 20% and 80%, respectively. If it rains, action 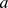 leads to 0 offspring for any given individual and action 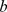 leads to 3 offspring; if it shines, the reverse occurs and action 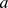 leads to 3 offspring while action 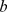 leads to 0 offspring. From an individual's perspective, choosing 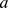 will lead to more reproductive success on average given the higher likelihood of sunshine. However, if all individuals in the population behaved in this manner, and rain or sunshine occurred for all individuals at the same time, the first time that a negative environment appears, the entire population of individuals that always choose 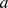 will become extinct. If we assume that offspring behave identically to their parents (perfect transmission of traits across generations), the behavior “always choose 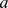
” cannot survive over time. In fact, we show below that the behavior with the highest reproductive success over time in this very specialized example is to randomize between 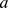 and 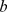 using the same probability as the probability of sunshine, 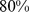; the group of individuals exhibiting this probability-matching behavior achieves the maximum possible growth rate. As a result, it appears as though selection operates at the group level and that this group—all individuals 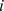 who randomize their actions with probability 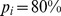
—is the fittest.

The key to this outcome is the fact that the reproductive risk facing all individuals in the population, rain or sunshine, is perfectly correlated, which we refer to as systematic risk. If we had assumed, instead, that reproductive risk was idiosyncratic–that the state of nature is independently and identically distributed (IID) for each individual–then the evolutionarily dominant strategy is, in fact, the purely “selfish” one in which 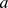 is chosen all the time.

This framework demonstrates that a separate theory of group selection is not strictly necessary to explain observed phenomena such as altruism and cooperation. But our results also show that the notion of group selection does capture a unique aspect of evolution—selection with correlated reproductive risk–that may be sufficiently widespread and distinct to warrant a separate term for the phenomenon. There is no controversy around the fact that selection occurs at the genetic level because of the basic biology of reproduction. However, we show that selection can also appear to operate at coarser levels if environmental forces affect a particular subset of individuals in similar fashion, i.e., if their reproductive risks are highly correlated. We use the term “appear” intentionally because in our framework, selection does not occur at the level of the group, but the behavior that is evolutionarily dominant is consistent with some of the empirical implications of group selection.

By studying the impact of selection on behavior rather than on genes, we are able to derive evolutionary implications that cut across species, physiology, and genetic origins. In the same way that different magnifications of a microscope reveal different details of a specimen, applying evolutionary principles to behavioral variations leads to different insights that may be more relevant for economics, psychology, and behavioral ecology. Because evolution is essentially a passive “process of elimination” [37], selection and adaptation operate at all levels, from genes to populations, depending on the nature of the corresponding environmental challenges. Our focus on behavior as the object of selection is a different lens through which the effects of evolution may be studied.

In the remainder of this paper, we provide a review of the literature, followed by an introduction to the binary choice model with two systematic environmental factors. In this framework, we derive a behavioral adaptation to stochastic environments with systematic risk in which groups seem to be the unit of selection, but which is purely the result of natural selection operating on individuals in the population. We conclude with a brief discussion of the results. A generalization of the binary choice model to the multinomial case with multiple factors is provided in the Supplementary Information, as well as all proofs and derivations.

### Literature Review

The literature on evolution and behavior can be overwhelming, spanning the disciplines of evolutionary biology, ecology, evolutionary and social psychology, and economics. While a comprehensive survey is well beyond the scope of this paper, we attempt to provide a representative sampling of the many strands that are most relevant to our focus.

The role of stochastic environments in evolution has been investigated extensively by biologists and ecologists. Stochastic environments cause high genetic variation and, in the extreme cases, extinction [38]–[44]. Environmental uncertainty that is associated with stochasticity over time [45]–[47] or heterogeneity across space [48] can cause natural selection to favor a gene that randomizes its phenotypic expression. Gillespie and Guess [49] describe a heuristic method to study selection in random environments. Frank [50]–[52] analyzes how variability in reproductive success affects fitness and relates it to the geometric mean principle. Geometric-mean fitness has also appeared in the financial context as the “Kelly criterion” for maximizing the geometric growth rate of a portfolio [53]–[56]. However, the motivation for geometric-mean fitness in population biology is considerably more compelling than in financial investments because maximizing the geometric-mean return of a portfolio is optimal only for individuals with a very specific risk preference, i.e., those with logarithmic utility functions [54].

In the evolutionary biology literature, Maynard Smith [57] has developed the concept of an “evolutionarily stable strategy”, specific behaviors that survive over time by conferring reproductive advantages or “fitness”, typically measured by the rate of population growth. Using this notion of fitness, Fretwell [58], Cooper and Kaplan [59], and Frank and Slatkin [60] observe that randomizing behavior can be advantageous in the face of stochastic environmental conditions. The impact of variability in reproductive success among individuals in a population has been shown to yield a kind of risk aversion (which increases average reproductive success) and “bet-hedging” (which reduces the variance of reproductive success) [47], [61]–[67]. Frank and Slatkin [60] propose a framework that highlights the importance of correlations among individual reproductive success in determining the path of evolution.

Similar results have been derived in the behavioral ecology literature, in which the maximization of fitness via dynamic programming has been shown to yield several observed behaviors, including risk-sensitive foraging in non-human animal species [68]–[79] and seed dispersal strategies in plants [80], [81]. Recently, the neural basis of risk aversion has also received much attention as researchers discovered that the activity of specific neural substrates correlates with risk-taking and risk-averse behaviors [82]–[85].

The relationship between risk-spreading behavior and kin selection has also been considered. Yoshimura and Clark [86] show that the risk-spreading polymorphism, in which a given genotype consists of a mixture of two or more forms each employing different behavioral strategies [48], [59], makes sense only for groups. Yoshimura and Jansen [87] argue that risk-spreading adaptation is a form of kin selection [59], and the strategies of kin can be very important in stochastic environments even if there are no interactions at all. Mcnamara [88] introduces the profile of a strategy and relates the geometric mean fitness to a deterministic game.

In the economics literature, evolutionary principles were first introduced to understand cooperation and altruism [89]–[91], and evolutionary game theory is considered a foundation for altruistic behavior [17], [92]. Evolutionary models of behavior are especially important for economists in resolving conflicts between individual rationality and human behavior [14], [18], including attitudes toward risk and utility functions [16], [17], [93]–[95], time preference [96]–[100], financial markets and firm selection [19], [20], [101], [102], and the economic analysis of social institutions [103]–[105].

Evolutionary models of behavior have also been used to justify the existence of utility functions and to derive implications for their functional form [16], [95], [106] (see Robson [18] and Robson and Samuelson [99] for comprehensive reviews of this literature). For example, Robson [16] investigates expected and non-expected utility behaviors, and finds that randomized behavior may be optimal from a population perspective even though it is sub-optimal from an individual perspective [107], [108]. Robson [95] argues that the kind of predictable behavior capable of being captured by a utility function emerged naturally as an adaptive mechanism for individuals faced with repeated choices in a nonstationary environment. Robson and Samuelson [97] find that exponential discounting in utility functions is consistent with evolutionarily optimal growth of a population.

### Binary Choice Model with Systematic Risk

Consider a population of individuals that live for one period, produce a random number of offspring asexually, and then die. During their lives, individuals make only one decision: they choose from two actions, 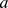 and 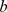, and this results in one of two corresponding random numbers of offspring, 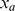 and 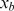. Now suppose that each individual chooses action 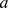 with some probability 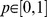 and action 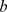 with probability 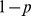, denoted by the Bernoulli variable 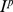, hence the number of offspring of an individual is given by the random variable:
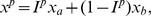



where
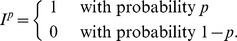



We shall henceforth refer to 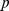 as the individual's *behavior* since it completely determines how the individual chooses between action 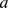 and 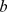. Note that 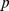 can be 0 or 1, hence we are not requiring individuals to randomize—this will be derived as a consequence of natural selection under certain conditions.

Now suppose that there are two independent environmental factors, 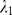 and 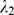, that determine reproductive success, and that 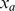 and 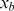 are both linear combinations of these two factors:
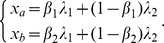



where 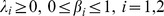. Examples of such factors are weather conditions, the availability of food, or the number of predators in the environment. Because these factors affect the fecundity of all individuals in the population, we refer to them as *systematic*, and we assume that:

(A1) 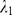 and 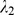 are independent random variables with some well-behaved distribution functions, such that 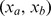 and 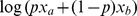 have finite moments up to order 2 for all 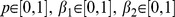, and

(A2) 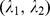 is IID over time and identical for all individuals in a given generation.

We shall henceforth refer to 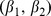 as an individual's *characteristics*. For each action, individuals are faced with a tradeoff between two positive environmental factors because of limited resources.

Under this framework, an individual is completely determined by his behavior 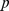 and characteristics 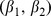. We shall henceforth refer to 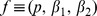 as an individual's *type*. We assume that offspring behave in a manner identical to their parents, i.e., they have the same characteristics 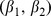, and choose between 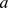 and 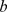 according to the same 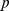, hence the population may be viewed as being comprised of different types indexed by the triplet 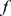. In other words, offspring from a type-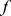 individual are also of the same type 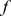, hence we are assuming perfect genetic transmission from one generation to the next (once a type 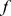, always a type 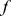). We also assume that the initial population contains an equal number of all types, which we normalize to be 1 each without loss of generality. In summary, an individual 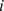 of type 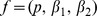 produces a random number of offspring:
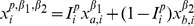
(1)


where
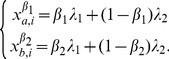
(2)


Now consider an initial population of individuals with different types. Suppose the total number of type 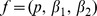 individuals in generation 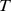 is 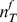. Under assumptions (A1)–(A2), it is easy to show that 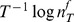 converges in probability to the log-geometric-average growth rate:
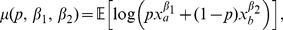
(3)


as the number of generations and the number of individuals in each generation increases without bound (we provide the proof for a more general case in the Supplementary Information). Define
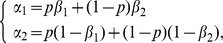



then 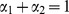, and (3) can be equivalently written as
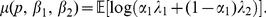
(4)


We shall henceforth refer to 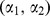 as the *factor loadings* of type-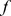 individuals, and (3) and (4) characterize the log-geometric-average growth rate of individuals as a function of their type 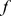 in terms of both behavior 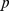 and characteristics 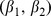.

Over time, individuals with the largest growth rate will dominate the population geometrically fast [14]. The optimal factor loading 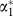 that maximizes (4) is given by:
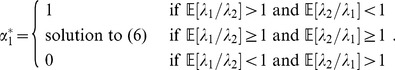
(5)


where 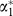 is defined implicitly in the second case of (5) by
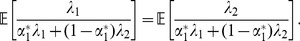
(6)


As a result, the growth-optimal type is 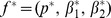, which is given explicitly in Table 1.

**Table 1:** Optimal type 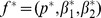 for the binary choice model

	Optimal characteristics	Optimal behavior
If 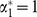	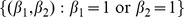	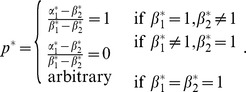
If 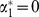	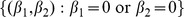	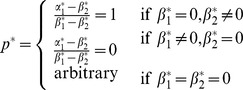
If 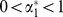	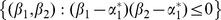	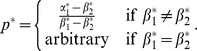

The optimal characteristics and associated optimal behaviors in Table 1 show that, when 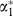 is 1 or 0, one of the factors, 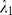 or 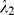, is significantly more important than the other, and the optimal strategy places all the weight on the more important factor. However, when 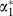 is strictly between 0 and 1, a combination of factors 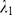 and 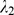 is necessary to achieve the maximum growth rate. Individual characteristics 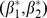 need to be distributed in such a way that one of the two choices of action puts more weight on one factor, while the other choice puts more weight on the other factor. Eventually, the behavior 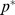 randomizes between the two choices and therefore achieves the optimal combination of factors. This is a generalization of the “adaptive coin-flipping” strategies of Cooper and Kaplan [59], who interpret this behavior as a form of altruism because individuals seem to be acting in the interest of the population at the expense of their own fitness. However, Grafen [107] provides a different interpretation by proposing an alternate measure of fitness, one that reflects the growth rate of survivors.

This result may be viewed as a primitive form of herding behavior—where all individuals in the population choose to act in the same manner—especially if the relative environmental factors, 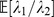 and 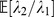, shift suddenly due to rapid environmental changes. To an outside observer, behaviors among individuals in this population may seem heterogenous before the shift, but will become increasingly similar after the shift, creating the appearance (but not the reality) of intentional coordination, communication, and synchronization. If the reproductive cycle is sufficiently short, this change in population-wide behavior may seem highly responsive to environmental changes, giving the impression that individuals are learning about their environment. This is indeed a form of learning, but it occurs at the population level, not at the individual level, and not within an individual's lifespan.

### Individually Optimal versus Group Optimal Behavior

It is instructive to compare the optimal characteristics and behavior in Table 1 that maximize growth with the behavior that maximizes an individual's reproductive success. According to (1) and (2), the individually optimal behavior maximizes
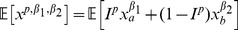


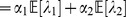



over 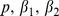. Therefore, the individually optimal factor loading, denoted by 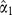, is simply:
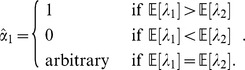



Given a particular environment 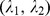, the individually optimal behavior depends only on the expectation of two factors, and this selfish behavior is generally sub-optimal for the group.

In contrast, individuals of type 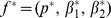 described in Table 1 are optimal in the group sense, attaining the maximum growth rate as a group by behaving differently than the individually optimal behavior. We shall refer to 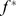 henceforth as the *growth-optimal* behavior to underscore the fact that it is optimal from the population perspective, not necessarily from the individual's perspective. This provides a prototype of group selection as a consequence of stochastic environments with systematic risk. We define *groups* to be individuals with the same characteristics. More precisely, in our model, individuals with the same 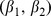 are considered a group. Nature selects the groups with optimal characteristics 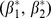, and 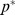 is a reflection of different behaviors for each group.

Like altruism, cooperation, trust, and other behaviors that do not immediately benefit the individual, the growth-optimal characteristics and behaviors derived in our framework flourish because they allow these individuals to pass through the filter of natural selection. However, unlike theories of group selection that are based on sexual reproduction and genetic distance, our version of group selection is based on behavior itself. Those individuals with types other than 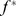 will not reproduce as quickly, hence from an evolutionary biologist's perspective, group selection is operating at the level of those individuals with characteristics 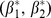 and behaving according to 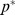. Of course, we cannot measure all forms of characteristics and behavior as readily as we can measure genetic make-up, but in the stark case of the binary choice model, it is clear that selection can and does occur according to groups defined by characteristics and behavior.

The Supplementary Information contains a generalization of the binary choice model to multinomial choices with multiple environmental factors. In general, it is possible that the optimal growth rate 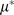 corresponds to multiple groups, and each group corresponds to multiple optimal behaviors. In terms of group selection, this fact means that several different groups—each defined by a specific combination of characteristics—could simultaneously be optimal from an evolutionary perspective. Within each group, natural selection will determine the behavior that achieves the optimal growth rate. The optimal behavior is not necessarily unique for each group. Also, the optimal behaviors for different groups might overlap.

### A Numerical Example

Consider an island that is isolated from the rest of the world, and suppose 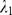 is a measure of weather conditions of the local environment, and 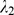 is a measure of the local environment's topography where, without loss of generality, we assume that larger values of each factor are more conducive to reproductive success. Moreover, 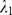 and 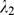 are independent random variables and described by:
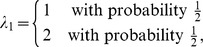


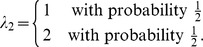



An individual on this island lives for one period, has one opportunity to choose one of two actions—farming (action 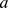) or mining (action 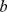)—which determines its reproductive success, and then dies immediately after reproduction. The number of offspring is given by 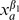 if action 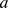 is chosen and 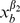 if 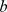 is chosen, where 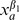 and 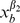 are given by:
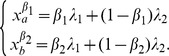



Here, 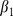 captures an individual's farming ability as determined by the two factors, weather and topography; 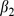 captures an individual's mining ability as determined by the same two factors. According to Table 1, the optimal factor loadings are ..., which indicates that individuals should have a balanced exposure to both weather and topography. The optimal characteristics are
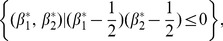
(7)


and each group is associated with the optimal behavior:
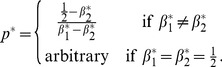
(8)


For example, 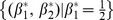 is an optimal group associated with 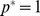. These are individuals who can perfectly balance the output of farming with respect to weather and topography. Therefore, they choose farming with probability 1 and appear as a “group” of farmers.

On the other hand, 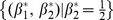 is another optimal group, but associated with the optimal behavior 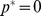. These individuals can perfectly balance the output of mining with respect to weather and topography. Therefore, they choose mining with probability 1 and appear as a “group” of miners.

Finally, there are also other optimal groups, described by (7) in general, in which individuals randomize their choices between farming and mining according to (8), to achieve the optimal exposure to weather and topography.

**Figure 1:**
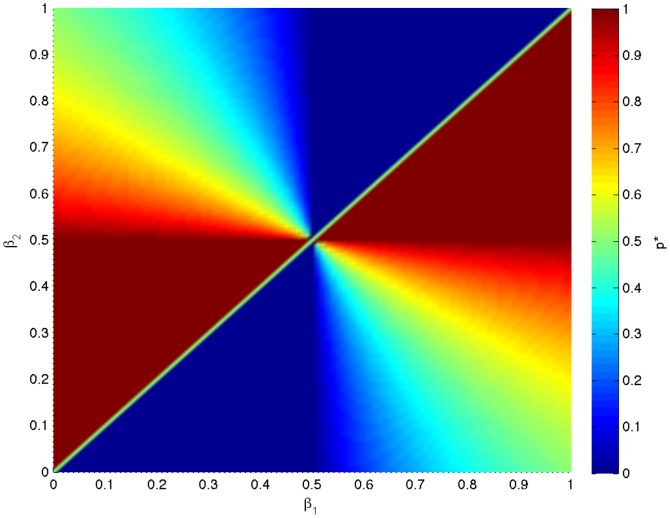
The optimal behavior for each group in the numerical example of group selection.

**Figure 2:**
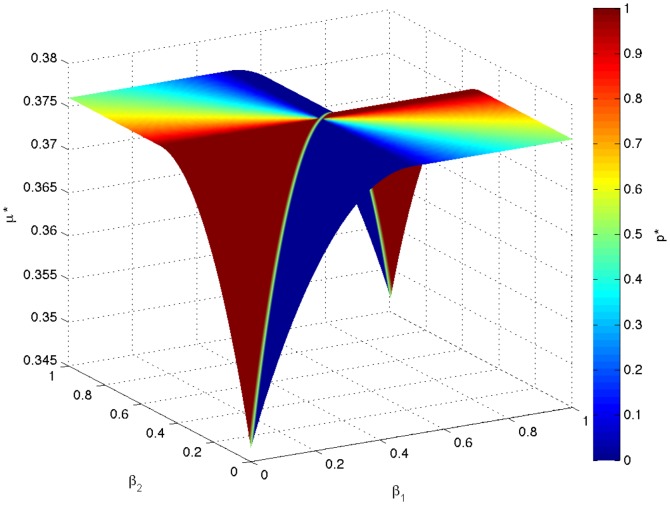
The optimal log-geometric-average growth rate for each group in the numerical example of group selection.

Figure 1 shows the optimal behavior for each group. The optimal groups described by (7) correspond to the upper-left and lower-right blocks. Randomized behaviors are optimal for these groups. Interestingly, all the sub-optimal groups (upper-right and lower-left blocks) correspond to deterministic behaviors (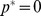 or 1) except when 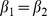. Figure 2 shows the optimal log-geometric-average growth rate for each group. It is clear that all groups described by (7) have the largest growth rate.

We can see that multiple optimal groups co-exist through natural selection, and within each group, individuals share the same characteristics. A particular behavior must be paired with a particular set of characteristics to achieve the optimal growth rate. Note that the individuals in (7) are optimal only in the group sense. As a single entity, a group possesses survival benefits above and beyond an individual, and in our framework, these benefits arise purely from stochastic environments with systematic risk.

The usual notion of group selection in the evolutionary biology literature is that natural selection acts at the level of the group instead of at the more conventional level of the individual, and interaction among members within each group is much more frequent than interaction among individuals across groups. In this case, similar individuals are usually clustered geographically. However, in our model, individuals do not interact at all, nevertheless, the fact that individuals with the same behavior generate offspring with like behavior makes them more likely to cluster geographically and appear as a “group”. In addition, imagine that the environment (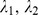) experiences a sudden shift. To an outside observer, behaviors among individuals in this population will become increasingly similar after the shift, creating the appearance—but not the reality—of intentional coordination, communication, and synchronization.

Here we use the phrase “appear as a group” because our derived behavior is not strictly the same as group selection as defined in the evolutionary biology literature. Instead, we show that behavior which is evolutionarily dominant through the traditional mechanism of natural selection is consistent with the implications of group selection. We purposefully model individuals in our population as mindless creatures engaging in random choices to demonstrate that group-like selection can arise purely through common factors in reproductive success. If we include more complex features such as sexual reproduction, limited resources, and competitive/cooperative interactions among individuals, even more sophisticated group dynamics can be generated.

## Discussion

Many species have a social structure in which individuals form groups and the aggregation of individuals promotes the fitness of group members. When selection for a biological trait in such populations depends on the difference between groups rather than individual differences within a group, it is described as “group selection” in evolutionary biology.

The debate surrounding genetic, kin, and group selection began over four decades ago, but has recently become more animated thanks to Nowak et al. [30], who challenge inclusive fitness theory in the study of social evolution by arguing that it is not a constructive theory that allows a useful mathematical analysis of evolutionary processes. Moreover, they conclude that inclusive fitness is neither useful nor necessary to explain the evolution of eusociality or other phenomena. However, this view was sharply criticized by a flurry of responses by many leading evolutionary biologists [35], [109]–[112], who observe that the more general inclusive fitness theory has stimulated the extensive empirical literature over the past 40 years in the fields of behavioral and evolutionary ecology [35], and that kin selection is a strong, vibrant theory that forms the basis for our understanding of how social behavior has evolved [110].

On the other hand, a significant amount of research suggests that group selection and kin selection (inclusive fitness) are essentially one process [28], [ 29], [ 31], [ 113]–[115], both seeking to characterize the genetic structure of a population but in different ways. These authors argue that it is now time to step back from the details of the specific arguments and consider the more general question of how evolution works in structured populations. This line of inquiry has the potential to generate insights beyond areas to which it has traditionally been applied [115].

Instead of entering into this debate, we propose to reconcile these opposing perspectives by studying the impact of selection on behavior and deriving evolutionary implications that cut across species, physiology, and genetic origins. As a direct consequence of this behavioral approach, we have shown that what appears to be group selection may, in fact, simply be the consequence of natural selection occurring in stochastic environments with reproductive risks that are correlated across individuals. In particular, we provide an evolutionary model with population dynamics for the simplest form of behavior, a binary choice, and derive the implications of selection on the behavior of individuals that share certain characteristics. Not surprisingly, individuals with similar characteristics experience similar selective pressures, hence evolution in stochastic environments with systematic risk can generate empirical phenomena that are consistent with group selection. In fact, Nature does select for “groups” of individuals with optimal characteristics and the optimal behavior within selected groups is simply a reflection of optimality of the characteristics of that group with respect to the given environment. Moreover, it is possible that multiple groups achieve the same optimal growth rate but through different means, i.e., many combinations of behavior and characteristics can be optimal, leading to considerable variation in the types of behavior and characteristics in the population.

Hamilton's great insight was that individual fitness is not maximized by social evolution; inclusive fitness is [2], [ 3]. The idea that something other than the individual organism could be the fitness-maximizing unit was completely revolutionary at the time and opened new research areas that are still being explored [111]. We have shown that, in addition to Hamilton's insight, individuals with highly correlated risks will appear to form “groups” in evolution, even if their actions are, in fact, totally autonomous, mindless, and, prior to natural selection, uniformly randomly distributed in the population. Although this result seems to eliminate the need for a separate theory of group selection, the unique and important evolutionary implications of multiple sources of correlated systematic risk suggest that a separate term for this phenomenon may be worthwhile.

## Acknowledgments

Research support from the MIT Laboratory for Financial Engineering is gratefully acknowledged. We thank the editor and the anonymous reviewers for helpful comments and suggestions, and Jayna Cummings for editorial assistance.

## Supporting Information

Text S1 **The general multinomial choice model and proofs of all the results in the main text are provided in this document.**
(PDF)
