# Supplementary material for: Group Selection as Behavioral Adaptation to Systematic Risk
Source: PLoS One. 2014 Oct 29;9(10):e110848. doi: 10.1371/journal.pone.0110848 (PMC4212975; doi:10.1371/journal.pone.0110848)
Supplement: Text S1 — The general multinomial choice model and proofs of all the results in the main text are provided in this document. (PDF) [file pone.0110848.s001.pdf]

Group Selection as Behavioral Adaptation to Systematic Risk: Supporting Text S1

Ruixun Zhang[1], Thomas J. Brennan[2], Andrew W. Lo[3,4,*]

1 MIT Department of Mathematics, Cambridge, Massachusetts, United States of America

2 Northwestern University School of Law, Chicago, Illinois, United States of America

3 MIT Sloan School of Management, CSAIL, and EECS, Cambridge, Massachusetts, United States of America

4 AlphaSimplex Group, LLC, Cambridge, Massachusetts, United States of America

∗ **E-mail: alo@mit.edu**

In this section, we first generalize the binary choice model to the general case where individuals face multinomial choices and the reproducibility is described by multiple environmental factors. Proofs for the main results are then provided.

1 The general model: multinomial choice with multiple factors

Consider a population of individuals that live for one period, produce a random number of offspring asexually, and then die. During their lives, individuals make only one decision: they choose from m actions $\{1, \cdots, m\}$, and this results in one of m corresponding random numbers of offspring $(x_1, \cdots, x_m)$. Suppose each individual chooses action i with probability p_i, for $i = 1, 2, \cdots, m$. Let $\mathbf{p} = (p_1, \cdots, p_m)$ be the probability vector that characterizes an individual's behavior. $\mathbf{p}$ satisfies the following conditions:

$$
\begin{aligned}
&0 \le p_i \le 1, \quad \forall i = 1, \cdots, m \\
&\sum_{i=1}^{m} p_i = 1.
\end{aligned}
$$

On the other hand, the environment is described by k factors $\boldsymbol{\lambda} = (\lambda_1, \cdots, \lambda_k)$. Let $\mathbf{B} = (\beta_{ij})_{m\times k}$ be the matrix of an individual's characteristics that satisfies the following conditions:

$$
\begin{aligned}
&0 \le \beta_{ij} \le 1, \quad \forall i = 1, \cdots, m; j = 1, \cdots, k \\
&\sum_{j=1}^{k} \beta_{ij} = 1, \quad \forall i = 1, \cdots, m.
\end{aligned}
$$

Note that the second condition is a direct generalization of the binary choice model, which means that each row of $\mathbf{B}$ sums to 1. This reflects the tradeoff between k environmental factors for each action.

In the multinomial choice model, an individual is characterized by both $\mathbf{p}$ and $\mathbf{B}$. The number of offspring for individual i with type $f = (\mathbf{p}, \mathbf{B})$ is:

$$
x_i^{\mathbf{p},\mathbf{B}} = I_{1,i}^{\mathbf{p}} x_{1,i}^{\mathbf{B}} + \cdots + I_{m,i}^{\mathbf{p}} x_{m,i}^{\mathbf{B}}
$$

where $(I_1^{\mathbf{p}}, \cdots, I_m^{\mathbf{p}})$ is the multinomial indicator variable with probability $\mathbf{p} = (p_1, \cdots, p_m)$:

$$(I_1^{\mathbf{p}}, \cdots, I_m^{\mathbf{p}}) = \begin{cases} (1, 0, \cdots, 0) & \text{with probability } p_1 \\ (0, 1, \cdots, 0) & \text{with probability } p_2 \\ \cdots \\ (0, 0, \cdots, 1) & \text{with probability } p_m, \end{cases}$$

and the number of offspring produced by taking each action is given by:

$$\begin{cases} x_{1,i}^{\mathbf{B}} = \beta_{11}\lambda_1 + \cdots + \beta_{1k}\lambda_k \\ \cdots \\ x_{m,i}^{\mathbf{B}} = \beta_{m1}\lambda_1 + \cdots + \beta_{mk}\lambda_k. \end{cases}$$

We assume that

(B1) $\lambda_1, \cdots, \lambda_k$ are independent random variables with some well-behaved distribution functions, such that $(x_1, \cdots, x_m)$ and $\log(p_1 x_1 + \cdots + p_m x_m)$ have finite moments up to order 2 for all $\mathbf{p} = (p_1, \cdots, p_m)$ and $\mathbf{B} = (\beta_{ij})_{m \times k}$, and

(B2) $(\lambda_1, \cdots, \lambda_k)$ is IID over time and identical for all individuals in a given generation.

Similar to the binary choice model, it is convenient to define factor loadings of type $f = (\mathbf{p}, \mathbf{B})$ individuals. Define $\boldsymbol{\alpha} = (\alpha_1, \cdots, \alpha_k) = \mathbf{pB}$:

$$(\alpha_1, \cdots, \alpha_k) = (p_1, \cdots, p_m) \begin{pmatrix} \beta_{11} & \cdots & \beta_{1k} \\ \vdots & \ddots & \vdots \\ \beta_{m1} & \cdots & \beta_{mk} \end{pmatrix}. \tag{1}$$

Note that $\alpha_1 + \cdots + \alpha_k = 1$ by definition.

Suppose that the total number of type f individuals in generation T is n_T^f, the following proposition gives us the log-geometric-average growth rate of type f in the general m-choice k-factor setting.

Proposition 1. *Under assumptions (B1)-(B2), as the number of generations and the number of individuals in each generation increases without bound, $T^{-1} \log n_T^f$ converges in probability to the log-geometric-average growth rate*

$$\mu(\mathbf{p}, \mathbf{B}) = \mathbb{E}\left[\log\left(\mathbf{pB}\boldsymbol{\lambda}'\right)\right] = \mathbb{E}\left[\log\left(\boldsymbol{\alpha}\boldsymbol{\lambda}'\right)\right]. \tag{2}$$

Proposition 1 characterizes the log-geometric-average growth rate as a function of type f. The next proposition gives the optimal type f^* that maximizes (2).

Proposition 2. *Under assumptions (B1)-(B2), the optimal factor loading $\boldsymbol{\alpha}^* = (\alpha_1^*, \cdots, \alpha_k^*)$ that maximizes (2) is given by:*

$$\boldsymbol{\alpha}^* = \begin{cases} (1, 0, \cdots, 0) & \textit{if } \mathbb{E}\left[\frac{\lambda_2}{\lambda_1}\right] < 1, \mathbb{E}\left[\frac{\lambda_3}{\lambda_1}\right] < 1, \cdots, \mathbb{E}\left[\frac{\lambda_k}{\lambda_1}\right] < 1 \\ (0, 1, \cdots, 0) & \textit{if } \mathbb{E}\left[\frac{\lambda_1}{\lambda_2}\right] < 1, \mathbb{E}\left[\frac{\lambda_3}{\lambda_2}\right] < 1, \cdots, \mathbb{E}\left[\frac{\lambda_k}{\lambda_2}\right] < 1 \\ \cdots \\ (0, 0, \cdots, 1) & \textit{if } \mathbb{E}\left[\frac{\lambda_1}{\lambda_k}\right] < 1, \mathbb{E}\left[\frac{\lambda_2}{\lambda_k}\right] < 1, \cdots, \mathbb{E}\left[\frac{\lambda_{k-1}}{\lambda_k}\right] < 1 \\ \textit{solution to (4)} & \textit{otherwise.} \end{cases} \tag{3}$$

In the last case, suppose without loss of generality $\boldsymbol{\alpha}^* = (\alpha_1^*, \cdots, \alpha_l^*, 0, \cdots, 0)$. That is, $\alpha_1, \cdots, \alpha_l$ are non-zero, $\alpha_{l+1}, \cdots, \alpha_k$ are zero. Then $\boldsymbol{\alpha}^*$ in the last case of (3) is defined implicitly by:

$$\mathbb{E}\left[\frac{\lambda_1}{\alpha_1^*\lambda_1 + \cdots + \alpha_l^*\lambda_l}\right] = \cdots = \mathbb{E}\left[\frac{\lambda_l}{\alpha_1^*\lambda_1 + \cdots + \alpha_l^*\lambda_l}\right] = 1, \tag{4}$$

and $\boldsymbol{\alpha}^*$ satisfies:

$$\begin{cases} \mathbb{E}\left[\frac{\lambda_{l+1}}{\alpha_1^*\lambda_1 + \cdots + \alpha_l^*\lambda_l}\right] < 1 \\ \cdots \\ \mathbb{E}\left[\frac{\lambda_k}{\alpha_1^*\lambda_1 + \cdots + \alpha_l^*\lambda_l}\right] < 1. \end{cases} \tag{5}$$

As a result, the growth-optimal type $f^* = (\mathbf{p}^*, \mathbf{B}^*)$ is given by

$$\mathbf{p}^*\mathbf{B}^* = \boldsymbol{\alpha}^*.$$

Note that in Proposition 2, it is not possible to fully characterize $\boldsymbol{\alpha}^*$ simply by the ratios $\mathbb{E}[\lambda_i/\lambda_j]$. However, there is still a natural analogue to the binary choice model by (4) and (5). $\boldsymbol{\alpha}^* = (\alpha_1^*, \cdots, \alpha_l^*, 0, \cdots, 0)$ is optimal if and only if the expectation of any irrelevant factor divided by the optimal combination of factor is less than 1, and any factor in the optimal combination divided by the optimal combination is equal to 1. Intuitively, this means that any factor in the optimal combination is adding a useful degree of freedom.

When the number of factors is no more than the number of choices: $k \leq m$, the maximization actually takes place in the k dimensional space $(\alpha_1, \cdots, \alpha_k)$. Putting it another way, there might be multiple solutions for the original probabilities $(p_1, \cdots, p_m)$ that correspond to the same factor combinations $(\alpha_1, \cdots, \alpha_k)$, and therefore the same growth rates.

It is important to reduce the dimensionality on which maximization takes place. If the dimension of the optimization problem is very large, then in practice a population won't be large enough for there to be a reasonable initial representation of all possible types. The factor model reduces the dimensionality of the problem, however, and makes it considerably more tractable, and more possible for the population to exhibit characteristics that respond to all the lower dimensional range of environmental uncertainty.

2 Proof of Table 1

Note that by definition $\alpha_1^* = p^*\beta_1^* + (1-p^*)\beta_2^*$, so α_1^* is a convex combination of β_1^* and β_2^*. If $\alpha_1^* = 1$, at least one of β_1^* and β_2^* must be 1. If $\alpha_1^* = 0$, at least one of β_1^* and β_2^* must be 0. If $0 < \alpha_1^* < 1$, β_1^* and β_2^* cannot be both greater than or both smaller than α_1^*. p^* can be solved accordingly given α_1^*, β_1^*, and β_2^*.

3 Proof of Proposition 1

The total number of type $f = (\mathbf{p}, \mathbf{B})$ individuals in generation T is

$$n_T^f = \sum_{i=1}^{n_{T-1}^f} x_i^{\mathbf{p},\mathbf{B}} = \sum_{i=1}^{n_{T-1}^f} I_{1,i}^{\mathbf{p}} x_1^{\mathbf{B}} + \cdots + I_{m,i}^{\mathbf{p}} x_m^{\mathbf{B}} = n_{T-1}^f \left(\frac{1}{n_{T-1}^f} \sum_{i=1}^{n_{T-1}^f} I_{1,i}^{\mathbf{p}} x_1^{\mathbf{B}} + \cdots + I_{m,i}^{\mathbf{p}} x_m^{\mathbf{B}} \right).$$

As n^f_{T-1} increases without bound, by Law of Large Numbers, it converges in probability to

$$n^f_{T-1}\left(p_1 x^{\mathbf{B}}_1 + \cdots + p_m x^{\mathbf{B}}_m\right) = n^f_{T-1} \cdot \mathbf{pB}\boldsymbol{\lambda}'_T.$$

Through backward recursion, the total number of type $f = (\mathbf{p}, \mathbf{B})$ individuals in generation T is

$$n^f_T \overset{p}{=} n^f_0 \cdot \prod_{t=1}^{T} \mathbf{pB}\boldsymbol{\lambda}'_t = \exp\left(\sum_{t=1}^{T} \log\left(\mathbf{pB}\boldsymbol{\lambda}'_t\right)\right).$$

Therefore,

$$\frac{1}{T}\log n^f_T \overset{p}{=} \frac{1}{T}\sum_{t=1}^{T}\log\left(\mathbf{pB}\boldsymbol{\lambda}'_t\right) \overset{p}{\to} \mathbb{E}\left[\log\left(\mathbf{pB}\boldsymbol{\lambda}'_t\right)\right]$$

as T increases without bound. Here "$\overset{p}{=}$" denotes equality in probability and "$\overset{p}{\to}$" denotes convergence in probability.

4 Proof of Proposition 2

We first prove the following lemma and then prove Proposition 2.

Lemma 1. $(\alpha^*_1, \cdots, \alpha^*_k)$ *maximizes (2) if and only if*

$$\mathbb{E}\left[\frac{\alpha_1\lambda_1 + \cdots + \alpha_k\lambda_k}{\alpha^*_1\lambda_1 + \cdots + \alpha^*_k\lambda_k}\right] \le 1, \quad \forall(\alpha_1, \cdots, \alpha_k). \tag{6}$$

Proof of Lemma. Note that (2) is a concave function with respect to $\alpha_1, \cdots, \alpha_k$, so a local maximum is the global maximum. Now suppose $\boldsymbol{\alpha}^* = (\alpha^*_1, \cdots, \alpha^*_k)$ is a local maximum, then a necessary and sufficient condition is that if we move $\boldsymbol{\alpha}^*$ toward a direction of any $\boldsymbol{\alpha} = (\alpha_1, \cdots, \alpha_k)$, the growth rate decreases. Formally, let

$$\boldsymbol{\alpha}^\delta = (1-\delta)\boldsymbol{\alpha}^* + \delta\boldsymbol{\alpha}$$

where $\boldsymbol{\alpha}$ is arbitrary and $0 \le \delta \le 1$, and

$$\mu(\boldsymbol{\alpha}^\delta) = \mathbb{E}\left[\log\left(\left((1-\delta)\alpha^*_1 + \delta\alpha_1\right)\lambda_1 + \cdots + \left((1-\delta)\alpha^*_k + \delta\alpha_k\right)\lambda_k\right)\right].$$

Then,

$$\begin{aligned}
&\boldsymbol{\alpha}^* = (\alpha^*_1, \cdots, \alpha^*_k) \text{ maximizes (2)} \\
\Longleftrightarrow & \left.\frac{\partial \mu(\boldsymbol{\alpha}^\delta)}{\partial \delta}\right|_{\delta=0} \le 0, \text{ for any } \boldsymbol{\alpha} = (\alpha_1, \cdots, \alpha_k) \\
\Longleftrightarrow & \mathbb{E}\left[\frac{(\alpha_1 - \alpha^*_1)\lambda_1 + \cdots + (\alpha_k - \alpha^*_k)\lambda_k}{\alpha^*_1\lambda_1 + \cdots + \alpha^*_k\lambda_k}\right] \le 0, \text{ for any } \boldsymbol{\alpha} = (\alpha_1, \cdots, \alpha_k) \\
\Longleftrightarrow & \mathbb{E}\left[\frac{\alpha_1\lambda_1 + \cdots + \alpha_k\lambda_k}{\alpha^*_1\lambda_1 + \cdots + \alpha^*_k\lambda_k}\right] \le 1, \text{ for any } \boldsymbol{\alpha} = (\alpha_1, \cdots, \alpha_k)
\end{aligned}$$

which completes the proof of the lemma. □

The first k conditions in (3) follow directly from the lemma. As of the last case, note that $\alpha_1 = 1 - \alpha_2 - \cdots - \alpha_k$ and we can write $\mu(\cdot)$ as a function of $(\alpha_2, \cdots, \alpha_k)$. Therefore $\boldsymbol{\alpha}^*$ is given by the

following equations:

$$\begin{cases} \frac{\partial \mu(\alpha_2,\cdots,\alpha_k)}{\partial \alpha_2}\Big|_{\alpha_{l+1}=\cdots=\alpha_k=0} = 0 \\ \frac{\partial \mu(\alpha_2,\cdots,\alpha_k)}{\partial \alpha_3}\Big|_{\alpha_{l+1}=\cdots=\alpha_k=0} = 0 \\ \cdots \\ \frac{\partial \mu(\alpha_2,\cdots,\alpha_k)}{\partial \alpha_l}\Big|_{\alpha_{l+1}=\cdots=\alpha_k=0} = 0. \end{cases} \tag{7}$$

Also, the following partial derivatives must be negative:

$$\begin{cases} \frac{\partial \mu(\alpha_2,\cdots,\alpha_k)}{\partial \alpha_{l+1}}\Big|_{\boldsymbol{\alpha}^*} < 0 \\ \cdots \\ \frac{\partial \mu(\alpha_2,\cdots,\alpha_k)}{\partial \alpha_k}\Big|_{\boldsymbol{\alpha}^*} < 0. \end{cases} \tag{8}$$

(7) yields

$$\mathbb{E}\left[\frac{\lambda_1}{\alpha_1\lambda_1 + \cdots + \alpha_l\lambda_l}\right] = \cdots = \mathbb{E}\left[\frac{\lambda_l}{\alpha_1\lambda_1 + \cdots + \alpha_l\lambda_l}\right].$$

Suppose that the above value is C, then

$$1 = \mathbb{E}\left[\frac{\alpha_1\lambda_1 + \cdots + \alpha_l\lambda_l}{\alpha_1\lambda_1 + \cdots + \alpha_l\lambda_l}\right] = (\alpha_1 + \cdots + \alpha_l)C = C.$$

(8) yields

$$\mathbb{E}\left[\frac{\lambda_j}{\alpha_1\lambda_1 + \cdots + \alpha_l\lambda_l}\right] < \mathbb{E}\left[\frac{\lambda_1}{\alpha_1\lambda_1 + \cdots + \alpha_l\lambda_l}\right] = 1$$

for $j = l+1, l+2, \cdots, k$. which completes the proof.
